# Overexpression of SKA Complex Is Associated With Poor Prognosis in Gliomas

**DOI:** 10.3389/fneur.2021.755681

**Published:** 2022-01-13

**Authors:** Shoukai Yu

**Affiliations:** Hongqiao International Institute of Medicine, Shanghai Tongren Hospital and Clinical Research Institute, Shanghai Jiao Tong University School of Medicine, Shanghai, China

**Keywords:** gliomas, SKA complex, prognosis, cell cycle, rare malignant tumor

## Abstract

The spindle and kinetochore-associated complex is composed of three members: SKA1, SKA2, and SKA3. It is necessary for stabilizing spindle microtubules attaching to kinetochore (KT) in the middle stage of mitosis. The SKA complex is associated with poor prognosis in several human cancers. However, the role of SKA complex in rare malignant diseases, such as gliomas, has not been fully investigated. We investigated several databases, including Oncomine, UALCAN, and cBioPortal to explore the expression profile and prognostic significance of SKA complex in patients with gliomas. Gene ontology and Kyoto Encyclopedia of Genes and Genome pathways were used to analyze the potential enriched pathways. The genes co-expressed with SKA complex were identified and used for developing a protein-protein interaction (PPI) network using the STRING database. We found a significant overexpression of the mRNA levels of SKA1, SKA2, and SKA3 in patients with glioma patients. Higher expression of SKA1 and SKA3, but not SKA2, was significantly correlated with shorter overall survival of patients with glioma. In glioma, SKA complex was found to be involved in nuclear division, chromosome segregation, and DNA replication. The results of PPI network identified 10 hub genes (CCNB2, UBE2C, BUB1B, TPX2, CCNA2, CCNB1, MELK, TOP2A, PBK, and KIF11), all of which were overexpressed and negatively associated with prognosis of patients with glioma. In conclusion, our study sheds new insights into the biological role and prognostic significance of SKA complex in glioma.

## Introduction

Gliomas encompass both lower-grade gliomas (LGGs) and glioblastoma multiforme (GBM) (1). According to World Health Organization (WHO), gliomas can be divided into four grades (I-IV), and they are common primary malignant intracranial tumors in adults (2). Diffuse LGGs, including WHO Grades II and III astrocytomas and oligodendrogliomas, form a biologically heterogeneous group (3). GBM (WHO Grade IV) is the most common primary adult brain tumor and also one of the deadliest cancers (4). The significance of genomic and proteomic research on identifying novel biomarkers, improving the early diagnosis, prognosis, and development of new treatment for LGG and GBM has been emphasized (5, 6).

Spindle and kinetochore associated complex consists of three family members: SKA1, SKA2, and SKA3. These members stabilized spindle microtubules attaching to kinetochore (KT) in the middle stage of mitosis (7, 8). Recent evidence has suggested that the expression of SKA complex is dysregulated and closely related to prognosis in several human cancers, including breast cancer, cervical cancer, liver cancer, and lung cancer (9). In breast cancer, overexpression of SKA2 was found to be associated with poor prognosis. The changes in the expression of SKA2 and SKA 3 were found to significantly affect the proliferation, migration, and invasion of cancer cells (10, 11). Higher expression of SKA1 was also found to be significantly correlated with poor prognosis in hepatocellular carcinoma (HCC). However, the clinical value of SKA complex (SKA1, SKA2, and SKA3) as a prognostic biomarker in gliomas has not been investigated fully. In this study, we integrated a large number of public databases to study the expression of SKA complex, explore its relationship with a patient's prognosis, and identify various pathways regulated by SKA complex in glioma. Further understanding of the potential clinical applications of SKA complex for these rare malignant tumors will help to improve the understanding of the pathogenesis of glioma and in identifying novel prognostic biomarkers.

## Methods

### Analysis of Expression Patterns of SKA Complex in Gliomas

We used the ONCOMINE database to compare the mRNA expression levels of SKA complex in tumor tissues as compared to normal tissues of gliomas. ONCOMINE is a cancer microarray database and web-based data-mining platform to perform powerful genome-wide expression analyses. The analysis type was gliomas vs. normal groups. The data type was mRNA, and the thresholds parameters were *p* < 0.0001 and multiple change = 1.5.

UALCAN is a web portal for in-depth analyses of gene expression data of The Cancer Genome Atlas (TCGA) (http://ualcan.path.uab.edu). The gliomas samples (Table 1) are available from the TCGA database and the Genotype-Tissue Expression (GTEx) project. Gene Expression Profiling Interactive Analysis (GEPIA) is an open and straightforward database that investigates publicly available cancer transcriptome data, such as gene expression data of TCGA. Both of them (12, 13) were utilized to investigate the expression of SKA1, SKA2, and SKA3 in gliomas and normal samples. The samples were available from UCSC XENA (https://xenabrowser.net/datapages/) and were analyzed through Toil (14).

**Table 1:** Clinicopathological characteristics of gliomas patients from the TCGA database.

**Characteristic**	**Low SKA1**	**High SKA1**	***p***	**Low SKA2**	**High SKA2**	***p***	**Low SKA3**	**High SKA3**	***p***
*n*	348	348		348	348		348	348	
WHO grade, *n* (%)			<0.001			0.031			<0.001
G2	186 (29.3%)	38 (6%)		126 (19.8%)	98 (15.4%)		183 (28.8%)	41 (6.5%)	
G3	116 (18.3%)	127 (20%)		113 (17.8%)	130 (20.5%)		112 (17.6%)	131 (20.6%)	
G4	7 (1.1%)	161 (25.4%)		74 (11.7%)	94 (14.8%)		14 (2.2%)	154 (24.3%)	
IDH status, *n* (%)			<0.001			0.691			<0.001
WT	39 (5.7%)	207 (30.2%)		120 (17.5%)	126 (18.4%)		49 (7.1%)	197 (28.7%)	
Mut	305 (44.5%)	135 (19.7%)		223 (32.5%)	217 (31.6%)		295 (43%)	145 (21.1%)	
1p/19q codeletion, *n* (%)			<0.001			<0.001			<0.001
codel	137 (19.9%)	34 (4.9%)		113 (16.4%)	58 (8.4%)		117 (17%)	54 (7.8%)	
non-codel	210 (30.5%)	308 (44.7%)		235 (34.1%)	283 (41.1%)		230 (33.4%)	288 (41.8%)	
Age, meidan (IQR)	40 (32, 51)	53 (38, 63)	<0.001	46 (34.75, 59)	45 (34, 58)	0.708	39.5 (32, 51)	53 (38.75, 63)	<0.001

According to WHO definition, the patients were categorized into three groups (G2-G4). For our study, the data, which were available in the form of TPM (transcripts per million), were converted to log2 (TPM + 1) for the analysis. These RNAseq data were downloaded from TCGA in the form of FPKM (fragments per kilobase per million), and then transformed into TPM for further analyses. The analyses included the correlation between the expression of SKA genes and clinical characteristics and prognosis of patients with glioma. The differential expression of SKA mRNA was evaluated *via* box plots and *t*-tests. The ggplot2 package in R Programming (v3.6.3) was used to generate heatmaps.

### Analysis of cBioPortal

The cBio Cancer Genomics Portal (cBioPortal) is a publicly available, open-source platform to explore cancer genome data. cBioPortal (www.cbioportal.org) for cancer genomics provides the visualization, analysis, and available downloads of large-scale multiomic cancer data.

This study utilized the cBioPortal database to analyze mRNA levels of SKA members. The Z-scores of SKA genes were applied to measure their expressions. Specifically, RNASeqV2 data in cBioPortal database are processed and normalized using RSEM, and these data correspond to the results file from TCGA (RNA Seq V2 RSEM). RSEM is a widely used tool for quantifying gene and isoform abundances from RNA-Seq data.

### Evaluation of the Prognostic Value of SKA Gene Expression in Gliomas

The diagnostic potential of SKA complex for patients with gliomas was evaluated by the Receiver Operator Characteristic (ROC) curve. The differential expression of mRNA was plotted through box plots, and compared by *t*-test. The overall survival (OS) of patients with gliomas was estimated by Kaplan—Meier curves (K-M curves) using R packages. The K-M curves were generated to estimate the relationship between SKA complex expression and the OS of patients. The associations of SKA gene expression with clinical characteristics of gliomas were evaluated by Pearson correlation. Further examination using the clinical data from TCGA led to the identification of the diagnostic and prognostic biomarkers for gliomas.

### Analysis of SKA-Associated Genes and Pathways Dysregulated in Gliomas

Gene expression data of gliomas were downloaded from TCGA; data of 696 patients were included (15). Pearson correlation coefficients (|r| > 0.4 and *p* < 0.001) were applied to measure and identify the co-expressed genes with SKA genes. The potential biological functions and signaling pathways related to SKA genes were explored using the “clusterProfiler” package in R (16). The GO and KEGG analyses were conducted. For GO analysis, biological process (BP), cell composition (CC) and molecular function (MF) were applied with *p* < 0.05 as statistically significant.

### Identification of an Ska-Associated Protein-Protein Interaction (PPI) Network

The STRING database (http://string-db.org) was utilized to provide a critical assessment and integration of protein–protein interaction networks for SKA co-expressed genes. This network includes direct (physical) as well as indirect (functional) associations with a score greater than 0.7 as significant (17). Cytoscape 3.8 (http://www.cytoscape.org) and CytoHubba plug-in were implemented to identify the top eight hub genes (18–20). In addition, further statistical analysis (*t*-test and Rank sum test) and visualization were conducted by R packages, SPSS 20.0, and GraphPad Prism 7 (21–23) with *P* value <0.05 considered as significant.

For normally distributed measurement data, they are expressed as mean ± standard deviation (x ± s). For the intergroup comparison, *t*-test was performed. When comparing the values at different time points of the same individual, a paired *t*-test was applied. Rank sum test was applied to compare the measurement data groups when the normal distribution assumptions are, in fact, valid.

## Results

### The SKA Complex Subunits Are Overexpressed in Gliomas

**Figure 1:**
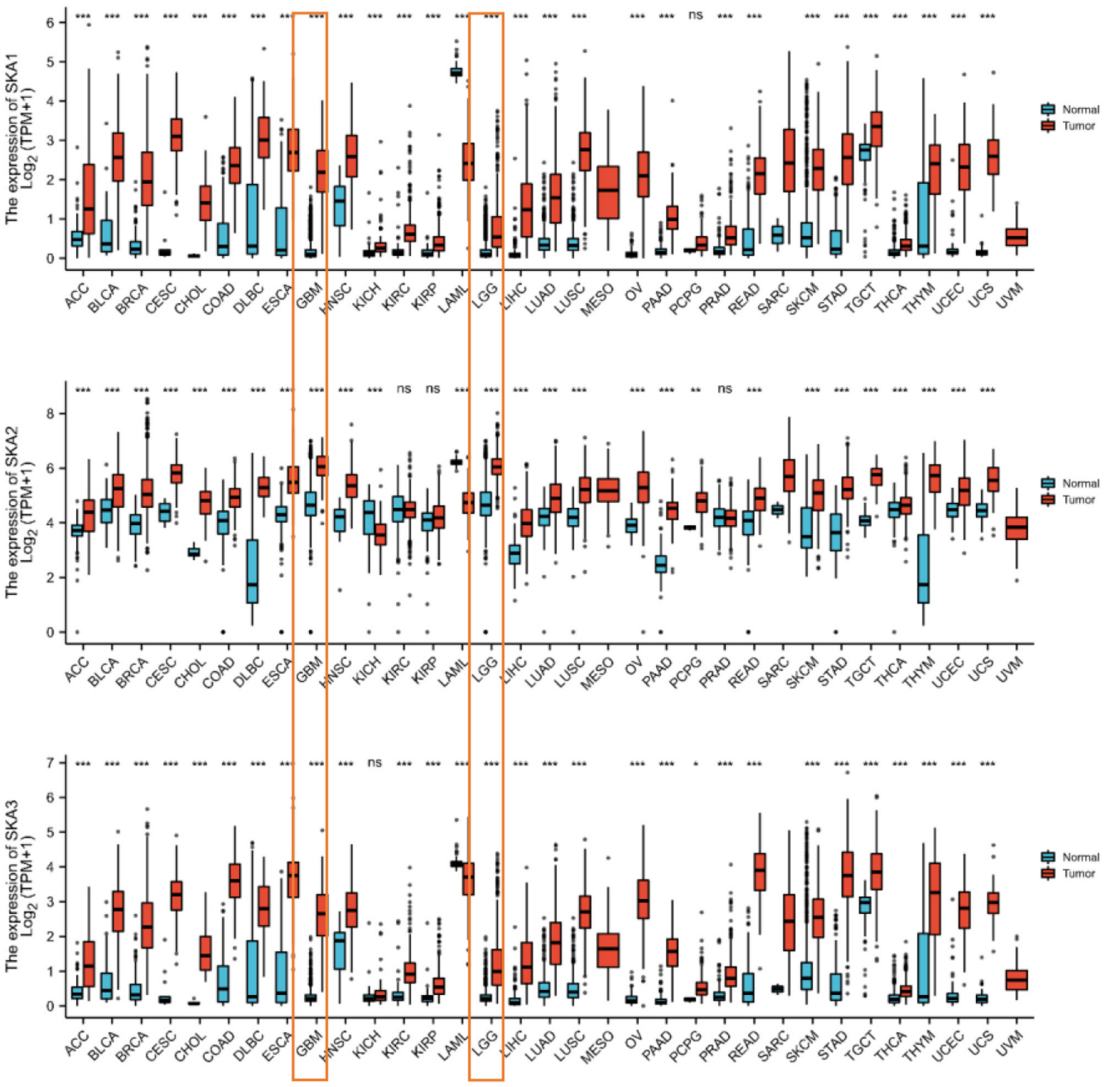
The expression of the SKA complex (SKA1, SKA2, and SKA3) genes in the tumor tissues and non-paired normal tissue in 33 human cancers. The expressions of LGG and GBM are boxed. *p < 0.05; **p < 0.01; ***p < 0.001; ns, not significant.

The mRNA levels of SKA complex between gliomas and normal tissues were compared using the ONCOMINE database. The mRNA levels of SKA1, SKA2, and SKA3 were significantly upregulated in gliomas (Figure 1). In addition, the mRNA levels of SKA1, SKA2, and SKA3 were significantly overexpressed in several other tumors, including breast, lung, colorectal, and kidney cancer (Figure 1).

**Figure 2:**
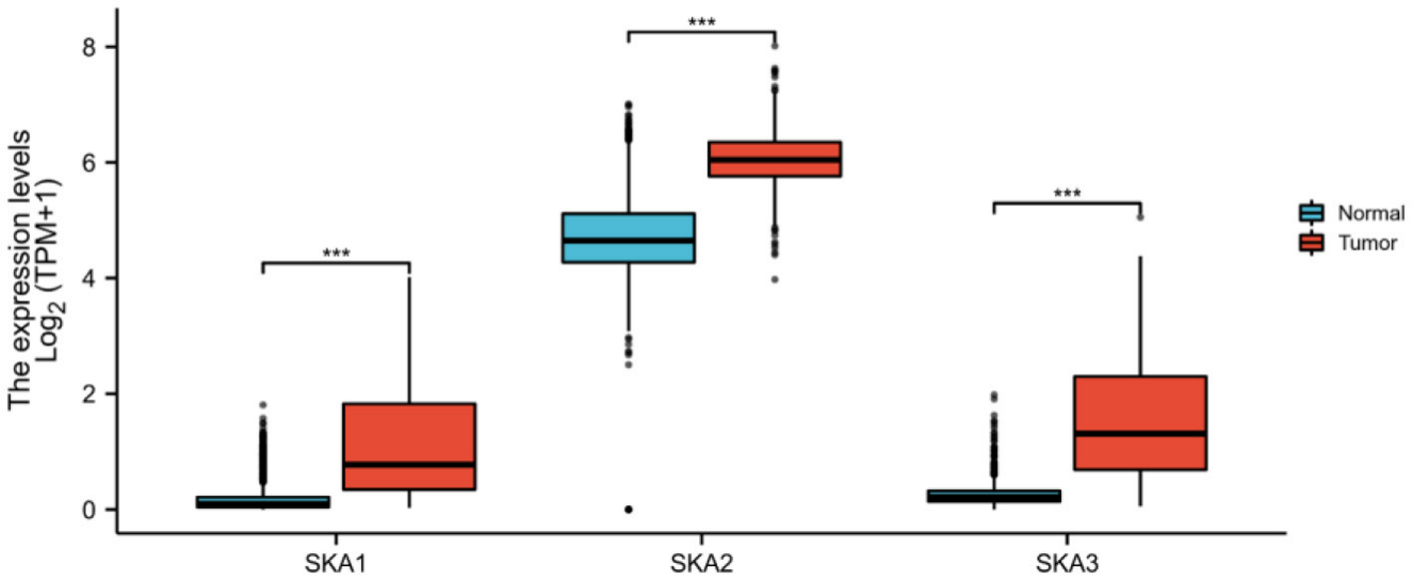
In the UALCAN database, the expression of SKA complex is significantly higher in the tumor tissues compared with normal tissue of patients with glioma (***p < 0.001).

**Figure 3:**
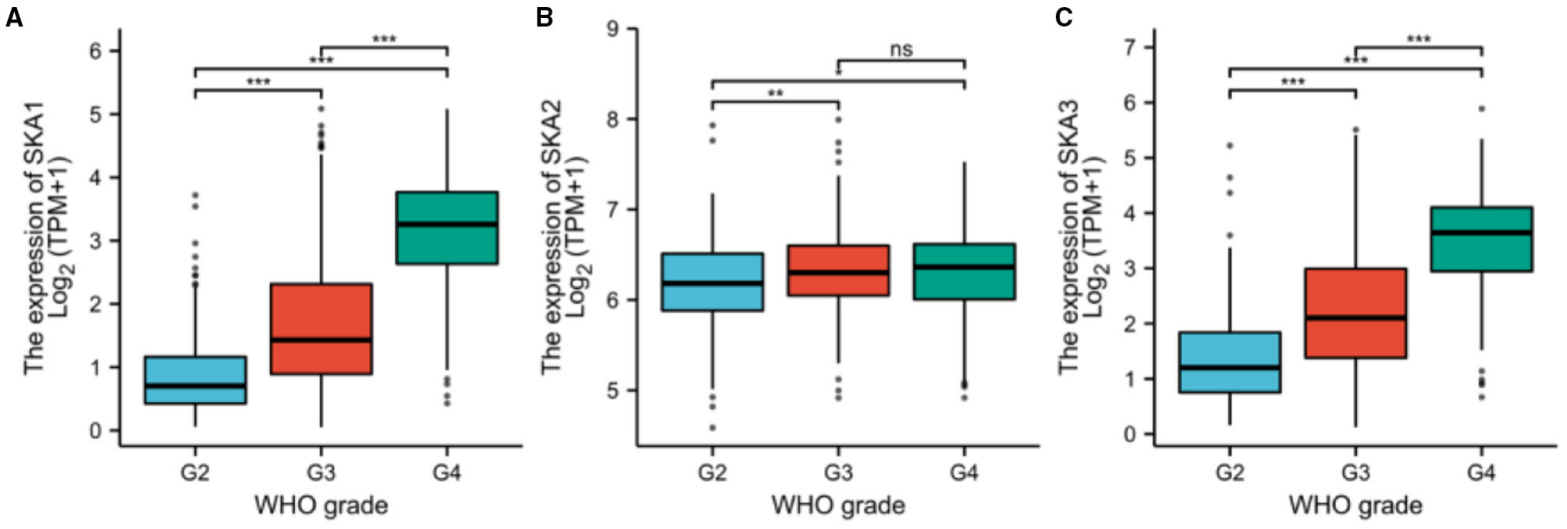
The expression of **(A)** SKA1, **(B)** SKA2, and **(C)** SKA3 in G2, G3, and G4 gliomas included in the UALCAN database. The grouping is based on WHO classification. **p < 0.01; ***p < 0.001; ns, not significant.

In order to identify whether the trend of SKA upregulation is recapitulated in other cancers (9, 10, 24), this study combined results from the UALCAN, cBioPortal, and TCGA databases. It has been observed that there is a statistically significant overexpression of the mRNA levels of SKA1, SKA2, and SKA3 in tumors as compared to normal tissues of patients with glioma included in the UALCAN database using Wilcoxon rank sum tests (*p* < 0.01; Figure 2). A significant overexpression of the mRNA levels of SKA1 and SKA3 was found in WHO Grade 4 (G4) as compared to G3 and G2 gliomas (Figure 3).

Furthermore, the expression of all three SKA complex subunits was significantly higher in WHO G3 tumors as compared to G2 tumors (Figure 3). The GEPIA database was also investigated to find any relationship between the expressions of SKA complex with OS in different grades of gliomas (Supplementary Figure 1). Interestingly, the expression of SKA1 and SKA3 was negatively correlated with OS in WHO G3 gliomas (*p* < 0.001 and *P* = 0.002, respectively; Supplementary Figure 1).

The analysis of samples from the cBioPortal database demonstrated that the change rates of mRNA levels were 0.2% for SKA1, 0.9% for SKA2, and 1.1% for SKA3 for LGG; 0.1% for SKA1, 0.3% for SKA2, and 0.9% for SKA3 for GBM, with increased expression for SKA3, correlated with gene amplification in a subset of the LGG and GBM tumor samples (Supplementary Figure 2).

### Diagnostic and Prognostic Values of SKA Complex in Gliomas

**Figure 4:**
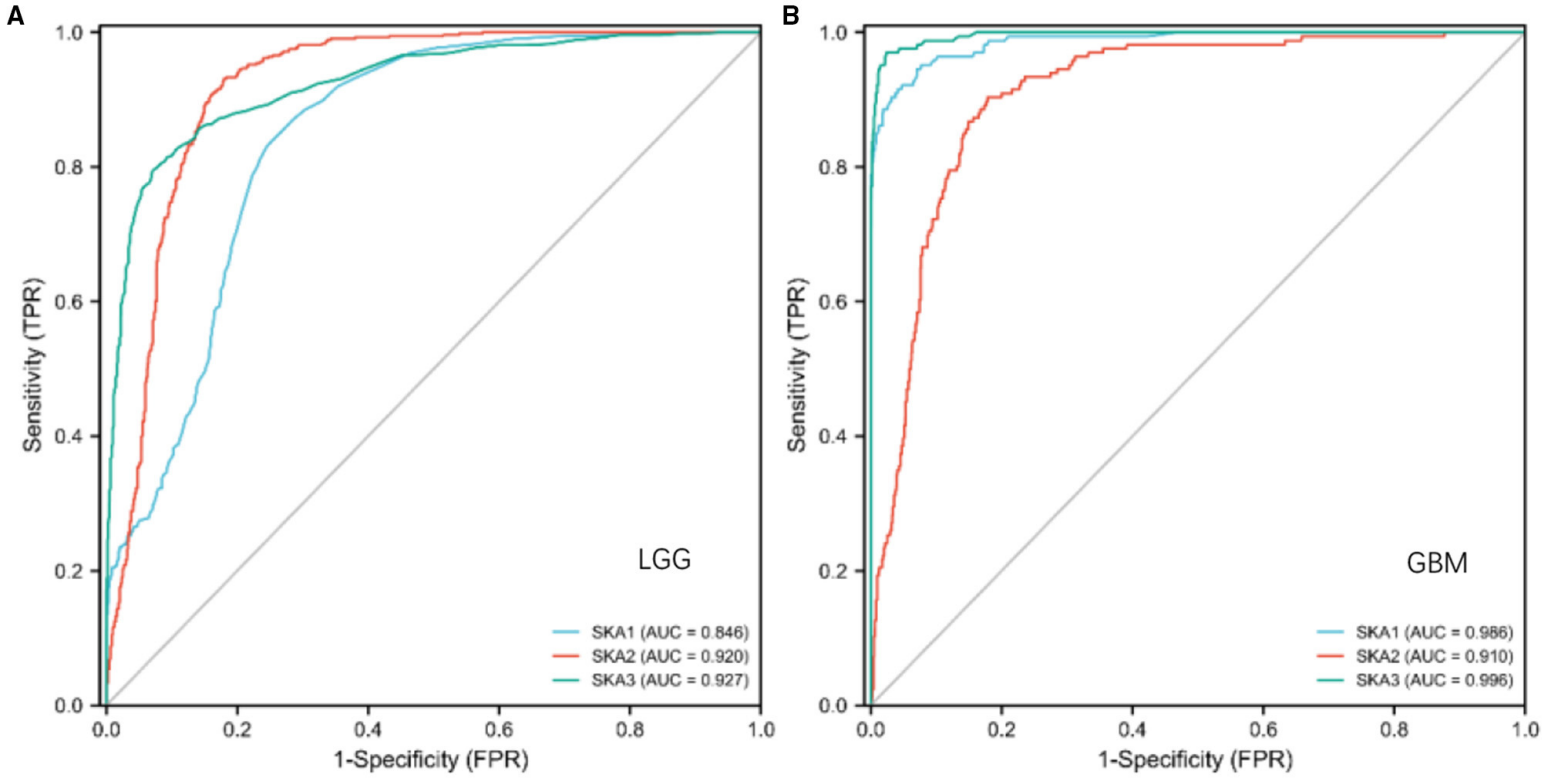
The diagnostic values of SKA complex in patients with gliomas. **(A)** LGG; **(B)** GBM.

**Figure 5:**
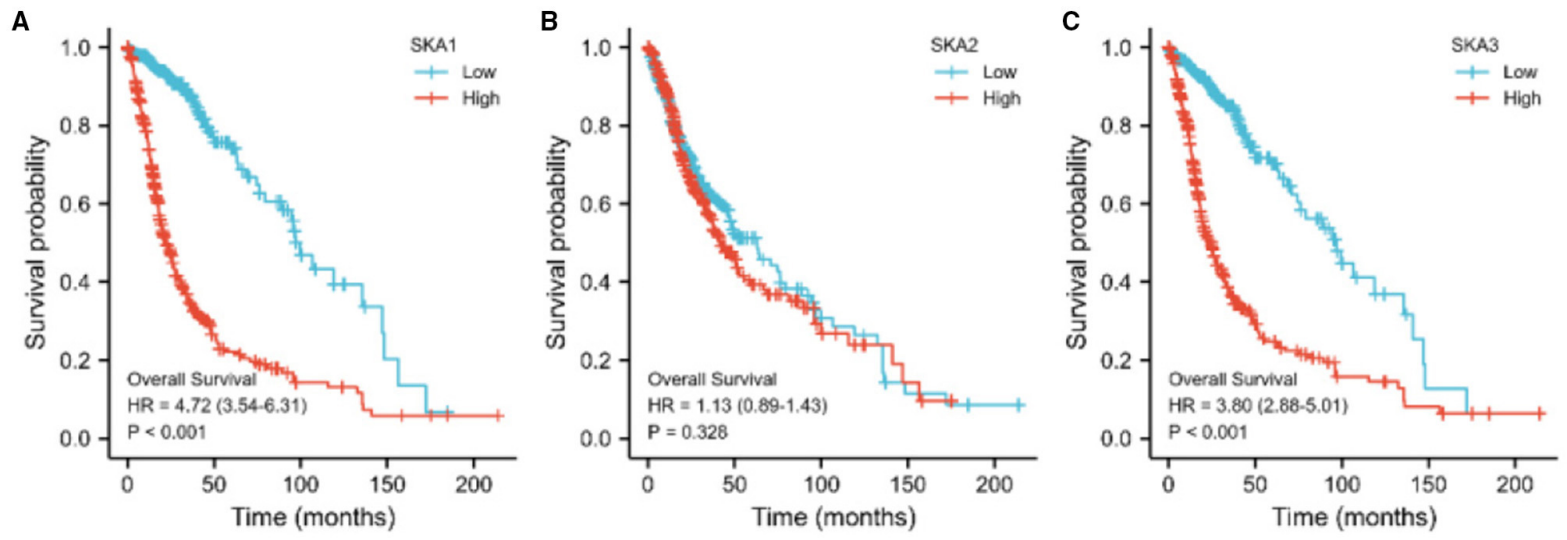
The relationship between the expression of **(A)** SKA1, **(B)** SKA2, and **(C)** SKA3 with OS of patients with gliomas in the GEPIA database.

We evaluated the diagnostic and prognostic values of SKA complex in gliomas using ROC curve analysis (Figure 4) and K-M curve analysis (Figure 5). The area under the curve (AUC) from the ROC curve was 0.846 for SKA1, 0.920 for SKA2, and.927 for SKA3 for patients with LGG (Figure 4A). The AUC from the ROC curve was.986 for SKA1, 0.910 for SKA2, and 0.996 for SKA3 for patients with GBM (Figure 4B). The expression data have been firstly ranked, and split into two groups (high and low) for SKA members separately (Supplementary Table 1). The significant difference has been identified between the two groups for all SKA members (Cox regression, *p* < 0.001). This significance not only confirmed this grouping strategy but also allows the largest possible sample size. Furthermore, the K-M curve analysis of SKA complex in patients with gliomas included in the GEPIA database demonstrated that the patients with gliomas who have higher expressions of SKA1 and SKA3 tend to have shorter OS than those with lower expression (*p* < 0.01; Figures 5A,C). The expression of SKA2 did not correlate with OS of patients with glioma (Figure 5B).

### Identification of Genes Co-expressed With SKA Complex

**Figure 6:**
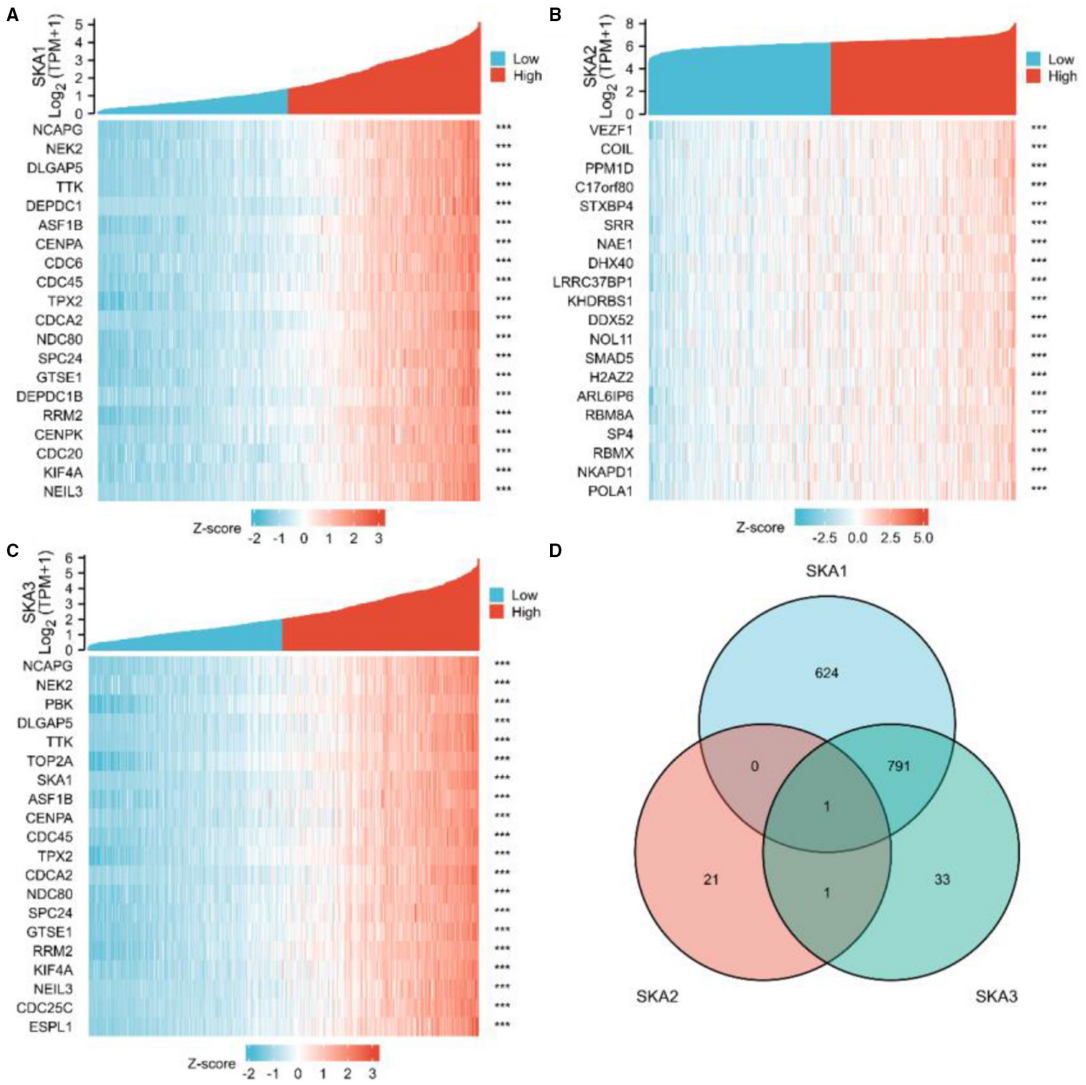
Identification of genes co-expressed with the SKA complex. Expression patterns from The Cancer Genome Atlas (TCGA) database are shown for the top 10 upregulated and the top 10 downregulated genes significantly correlated with the expression of SKA1 **(A)**, SKA2, **(B)**, and SKA3 **(C)** in gliomas. The intersection of co-expression patterns identifies the genes co-expressed with the SKA complex **(D)**. The threshold values are |r| >0.6 and *P*-value < 0.001.

We further investigated the role of SKA complex in gliomas by identifying genes co-expressed with SKA complex subunits. We found a significant correlation of the expression of 1,416 genes with SKA1, 23 genes with SKA2, and 826 genes with SKA3 when analyzed using the TCGA database (absolute correlation >0.6 and *p* < 0.001). Top 10 genes whose expression is positively and negatively correlated with SKA complex have been demonstrated in the heat-maps (Figures 6A–C). Interestingly, we found that only one gene co-expressed with all the three SKA subunits, as depicted in the Venn diagram (Figure 6D).

### GO and KEGG Analysis

To explore the biological functions of SKA complex, we performed GO and KEGG analyses on the co-expressed genes of SKA complex. GO analysis revealed that SKA-complex co-expressed genes were mainly enriched in nuclear division, chromosome segregation, and DNA replication in gliomas (Supplementary Figure 3). KEGG analysis revealed that SKA-complex co-expressed genes are mainly involved in the cell cycle, mismatch repair, and DNA replication (Supplementary Figure 3).

For additional insight into the role of the elevated SKA expression in gliomas, the results of GO and KEGG analyses reveal that SKA-complex co-expressed genes in gliomas were mainly enriched in the cell cycle and DNA replication pathways. We further identified hub genes by PPI network analysis, which predicted co-regulation of the SKA complex with DLGAP5, SF1, NEK2, H2AX, PBK, PTEN, ATRX, and DAXX (Supplementary Figures 4, 5). Each of these genes was verified to be overexpressed with the SKA subunit genes in gliomas. Our findings provide additional understanding of the mechanisms of gliomas and suggest an approach for biomarker discovery using publicly available resources.

Except ATRX, the overexpression of all other genes was found to be significantly correlated with shorter OS of patients with glioma (Supplementary Figure 4). We found an overexpression of DLGAP5, NEK2, and PBK, while the expression of SF1, PTEN, ATRX, and DAXX was downregulated in tumor tissues as compared to normal tissues of the patients with glioma (Supplementary Figure 5).

## Discussion

Our study demonstrates not only the diagnostic and prognostic relevance of SKA complex but also the biological pathways they regulate in gliomas. SKA complex, which consists of SKA1, SKA2, and SKA3, is essential for progression from metaphase to anaphase (25, 26). Previous studies have documented the overexpression of SKA complex in various human cancers. One of the studies reported that the overexpression of SKA2 was significantly associated with the clinical stage and lymph node metastasis (11). In gastric cancer, the expression of SKA2 was found to be regulated by miR-520a-3p, which acted as a tumor suppressor miRNA (27).

The overexpression of SKA1 has been found to be significantly associated with early recurrence and progression in several different cancer cells. The prognostic value and functional bioinformatics analysis of SKA1 also demonstrated that SKA1 might be a promising target for cancer gene therapy. The knockdown of SKA1 inhibited invasion and migration and invasion of tumor cells (24, 28). SKA2 was also found to promote proliferation and invasion of cancer cells (11, 29). Compared to SKA1 and SKA2, SKA3 is a relatively newly discovered gene, and its function related to anaphase transition is less explored. Analysis of the tumor characteristics of the patients (Table 1) suggested that SKA1 and SKA3 overexpression was correlated with the IDH status and patient age. Moreover, the expression of all three SKA genes was correlated with the WHO grade and 1p/19q codeletion (Table 1).

To the best of our knowledge, our studies report the potential prognostic utility of SKA complex in gliomas for the first time. Previous studies do report the potential clinical values of SKA complex in other human cancers (9, 11, 24). In this study, we found an overexpression of SKA complex in the tumor tissues of patients with gliomas. Furthermore, high mRNA levels of SKA1 and SKA3 complex were significantly associated with shorter OS of patients with glioma (Supplementary Table 1 and Figure 5). Therefore, SKA complex has the potential to serve as prognostic biomarkers in gliomas. The GO and KEGG results demonstrated that the co-expressed genes with SKA complex were enriched in nuclear division, chromosome segregation, and the DNA replication pathway. The results of PPI network identified eight hub genes: DLGAP5, SF1, NEK2, H2AX, PBK, PTEN, ATRX, and DAXX. This result is consistent with the previous research on other cancer biomarkers: CCNB2 (30), UBE2C (31), BUB1B (32–34), TPX2 (35), CCNA2 (36), CCNB1 (37, 38), MELK (35, 39), TOP2A (40–42), PBK, and KIF11. In GEPIA databases, these eight hub genes were all overexpressed and negatively associated with the prognosis of patients with gliomas.

In previous studies, several biomarkers associated with poor prognosis have been identified. Among them, eight potential biomarkers were selected from the GEPIA database: DLGAP5, SF1, NEK2, H2AX, PBK, PTEN, ATRX, and DAXX (34, 39, 43–47). DLGAP5 has a unique function in stabilizing spindle formation, and it is strongly expressed during all phases of mitosis. The combination of BUB1B, DLGAP5, and PINK1 can serve as a predictor of a poor outcome in adrenocortical tumors (34, 48). The overexpression of NEK2 has been found in various tumor types and cancer cell lines, and its expression is associated with rapid relapse and a poor outcome in multiple cancer types. DAXX is a highly conserved mammalian gene, and it is also a transcriptional co-repressor for various transcription factors, such as p53. DAXX plays an important role in many nuclear processes, such as transcription and cell cycle regulation (46). In the future, further experimental and clinical studies are suggested to confirm these findings and promote clinical application of biomarkers as an indicator of prognosis for gliomas. At the cellular level, further verification was required for the function of SKA complex.

There are eight genes included in the analysis: DLGAP5, SF1, NEK2, H2AX, PBK, PTEN, ATRX, and DAXX. The results demonstrate the relationship of the expression of representative 8 previously reported genes with OS of patients with gliomas (Supplementary Figure 4), and the expression patterns of these genes in tumor tissues and normal tissues of patients with glioma (Supplementary Figure 5) in the GEPIA database. These eight genes were identified to have the potential to serve as biomarkers for poor prognosis in gliomas. The expressions of these genes are significantly different between the tumor groups and the normal groups, except for H2AX.

Interestingly, the results identified SKA1 and SKA3, which are significantly correlated with overall survival, whereas SKA2 is not. Furthermore, survival of low and high SKA1 and SKA3 expressing patients differs in Grade 3 patients but not in Grade 2 and Grade 4 patients (Supplementary Figure 1). These significant results are based on the current samples, which indicate the important effect of SKA members in Grade 3 patients, especially for SKA1 and SKA3. The other non-significant results may be due to the limited sample size. More likely, SKA1 and SKA3 played a more crucial role compared to SKA2 in those identified pathways for gliomas. Compared to Grade 2 and Grade 4, the significance of SKA expression in Grade 3 suggests the importance of spindle and kinetochore in clinical stages. In addition, the biological and clinicopathological characteristics of SKA complex have also been changed during the influence on cancer progression.

Previous research showed that different SKA members have been significantly identified in different cancers (7, 8, 10, 24, 49). The member SKA1 was associated with alphafetoprotein (AFP), tumor size, and the TNM stage in patients with HCC and proliferation, the clinical stage and lymph node metastasis in NSCLC. The member SKA2 was associated with the proliferation, migration, and invasion of cancer cells in patients with breast cancer. The member SKA3 was associated with proliferation and migration of cancer cells and tumor growth in patients with cervical cancer. Based on the results (Figure 6D), there are much more related genes identified with SKA1 and SKA3, but less genes for SKA2 in gliomas. This could be an indication that SKA1 and SKA3 are better prognostic biomarkers for gliomas.

There are previous studies that focused on the role of SKA molecules in cell lines. For example, long non-coding RNA SPRY4-IT1 was shown to be associated with upregulation of SKA2 (50). However, these molecules were not identified in the current study based on our thresholds (absolute correlation >0.7; *P* value < 0.001). One possible explanation is that there are limited samples to identify the effect of related molecules. In summary, the mRNA level of SKA complex was significantly upregulated in gliomas. Moreover, the overexpression of SKA1 and SKA3 was significantly associated with poor prognosis of patients with gliomas. These results suggested that SKA complex has the potential to serve as one of the prognostic biomarkers and therapy targets for gliomas.

## Data Availability Statement

The datasets presented in this study can be found in online repositories. The names of the repository/repositories and accession number(s) can be found in the article/Supplementary Material.

## Ethics Statement

Ethical review and approval was not required for the study on human participants in accordance with the local legislation and institutional requirements. Written informed consent for participation was not required for this study in accordance with the national legislation and the institutional requirements.

## Author Contributions

SY conceived the study, carried out statistical analysis, and wrote and approved the manuscript.

## Funding

This work was supported by the Shanghai Pujiang Talent Program 19PJC085.

## Conflict of Interest

The author declares that the research was conducted in the absence of any commercial or financial relationships that could be construed as a potential conflict of interest.

## Publisher's Note

All claims expressed in this article are solely those of the authors and do not necessarily represent those of their affiliated organizations, or those of the publisher, the editors and the reviewers. Any product that may be evaluated in this article, or claim that may be made by its manufacturer, is not guaranteed or endorsed by the publisher.
