# Supplementary material for: Overexpression of SKA Complex Is Associated With Poor Prognosis in Gliomas
Source: Front Neurol. 2022 Jan 13;12:755681. doi: 10.3389/fneur.2021.755681 (PMC8791909; doi:10.3389/fneur.2021.755681)
Supplement: Supplementary file 1 [file Presentation_1.pdf]

Supplementary material

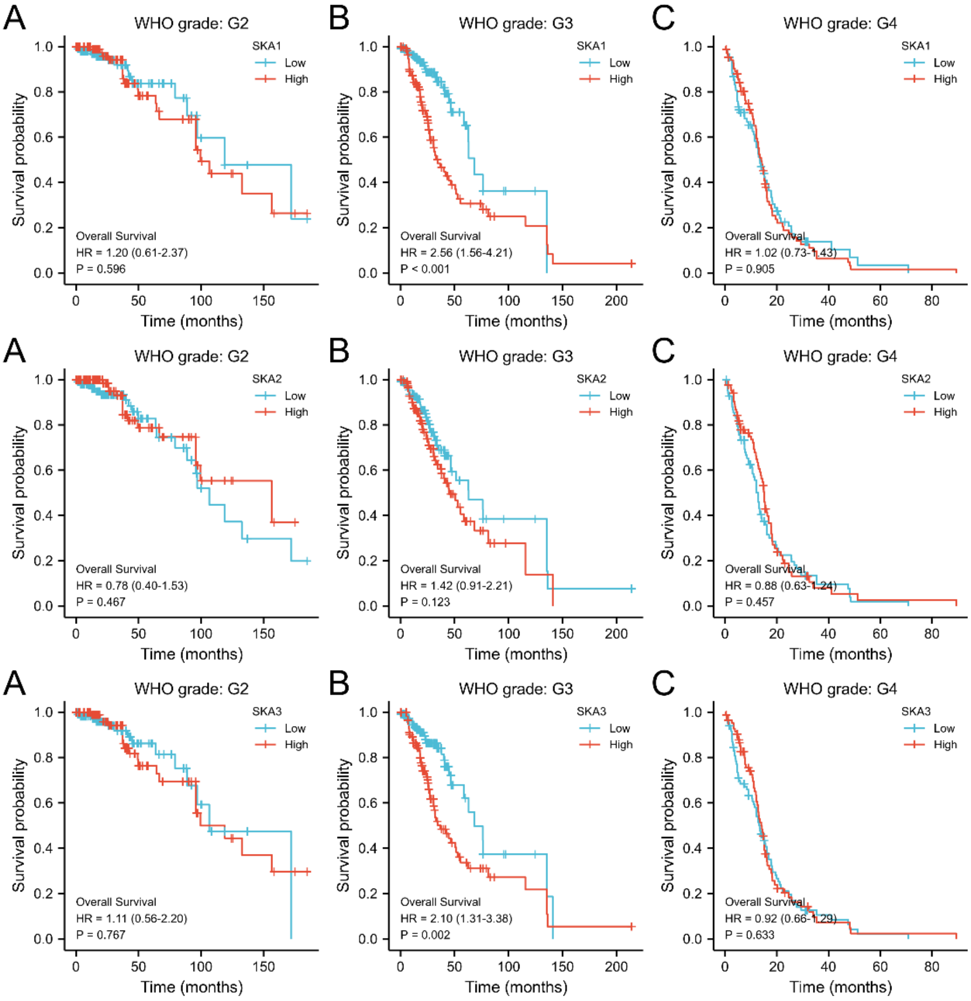

Figure S1: The relationship between the expression of SKA complex with OS according to different grades i.e. WHO G2, G3, and G4 of gliomas included in the GEPIA database. The top row is for SKA1; the middle row is for SKA2; and the bottom row is SKA3. (A) WHO Grade 2; (B) WHO Grade 3; (C) WHO Grade 4.

(A) LGG

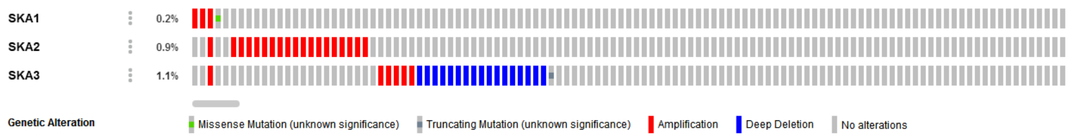

(B) GBM

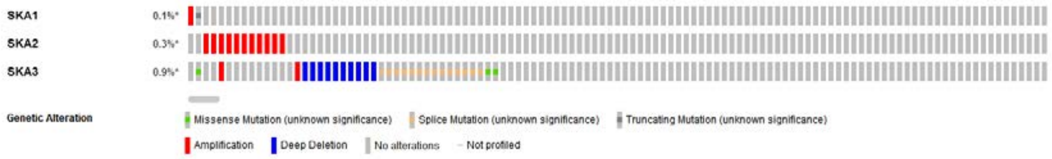

Figure S2 Heatmap of SKA complex expression in LGG (A) and GBM (B) patients. These heatmaps are shown for the expression of SKA1, SKA2, and SKA3 in tumor versus normal samples from the cBioPortal database. The amplifications status of the samples was determined by copy number analysis.

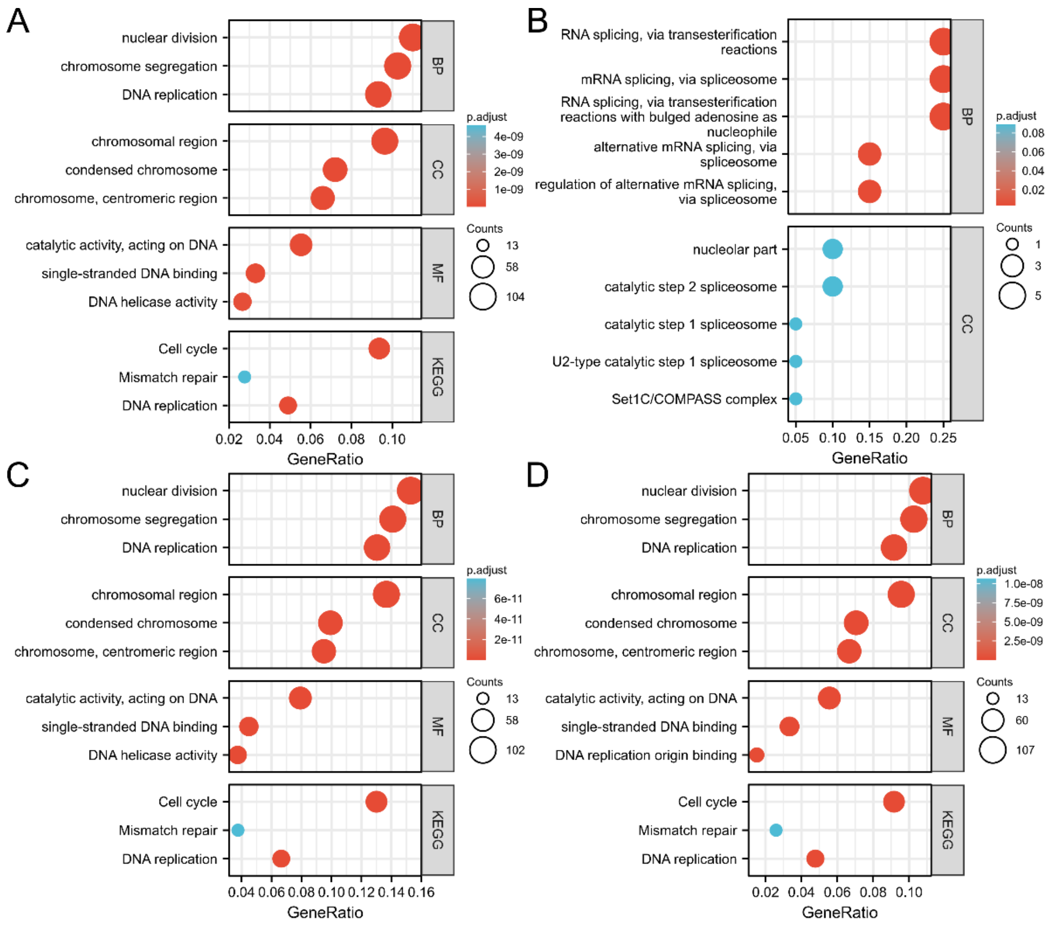

Figure S3: Gene ontology (GO) and Kyoto Encyclopedia of Genes and Genome (KEGG) analyses of genes co-expressed with the SKA complex in gliomas. GO categories for biological processes (BP), cellular composition (CC), and molecular functions (MF); and KEGG pathways (KEGG) are shown for genes correlated with SKA1 (A), SKA2 (B), SKA3 (C), and the union at all three subunits of the SKA complex (D).

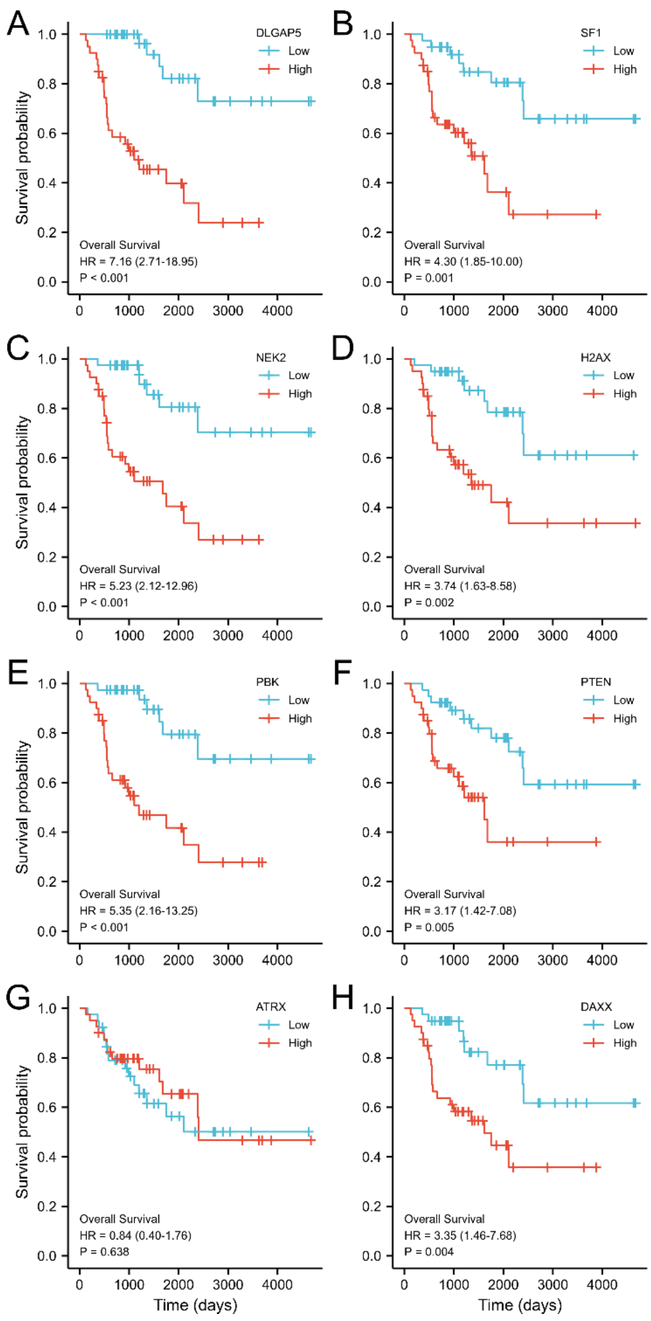

Figure S4: The relationship of the expression of representative 8 previously reported genes with OS of patients with gliomas in the GEPIA database. Eight genes included in the analysis are DLGAP5 (A), SF1 (B), NEK2 (C), H2AX (D), PBK (E), PTEN (F), ATRX (G), and DAXX (H).

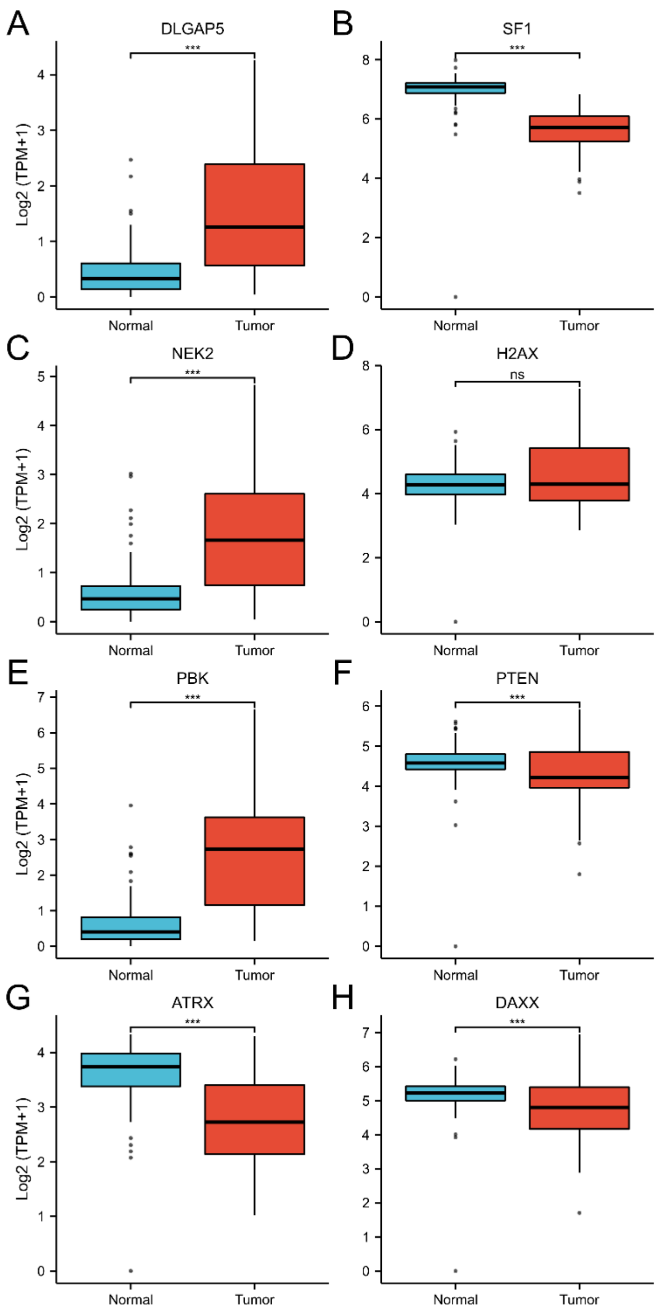

Figure S5: The expression patterns of representative 8 previously reported genes in tumor tissues and normal tissues of glioma patients in GEPIA database. These eight genes, DLGAP5 (A), SF1 (B), NEK2 (C), PBK (E), PTEN (F), ATRX (G), and DAXX (H), were previously reported to have the potential to serve as biomarkers for poor prognosis in gliomas. The expressions of these genes are significantly different between the tumor groups and the normal groups, except for H2AX (D).

Table 1 Clinicopathological characteristics of gliomas patients from the TCGA database.

Characteristic	Low SKA1	High SKA1	p	Low SKA2	High SKA2	p	Low SKA3	High SKA3	p
n	348	348		348	348		348	348	
WHO grade, n (%)			< 0.001			0.031			< 0.001
G2	186 (29.3%)	38 (6%)		126 (19.8%)	98 (15.4%)		183 (28.8%)	41 (6.5%)	
G3	116 (18.3%)	127 (20%)		113 (17.8%)	130 (20.5%)		112 (17.6%)	131 (20.6%)	
G4	7 (1.1%)	161 (25.4%)		74 (11.7%)	94 (14.8%)		14 (2.2%)	154 (24.3%)	
IDH status, n (%)			< 0.001			0.691			< 0.001
WT	39 (5.7%)	207 (30.2%)		120 (17.5%)	126 (18.4%)		49 (7.1%)	197 (28.7%)	
Mut	305 (44.5%)	135 (19.7%)		223 (32.5%)	217 (31.6%)		295 (43%)	145 (21.1%)	
1p/19q codeletion, n (%)			< 0.001			< 0.001			< 0.001
codel	137 (19.9%)	34 (4.9%)		113 (16.4%)	58 (8.4%)		117 (17%)	54 (7.8%)	
non-codel	210 (30.5%)	308 (44.7%)		235 (34.1%)	283 (41.1%)		230 (33.4%)	288 (41.8%)	
Age, meidan (IQR)	40 (32, 51)	53 (38, 63)	< 0.001	46 (34.75, 59)	45 (34, 58)	0.708	39.5 (32, 51)	53 (38.75, 63)	< 0.001
